# Program evaluation of a model to integrate internationally educated health professionals into clinical practice

**DOI:** 10.1186/1472-6920-13-140

**Published:** 2013-10-11

**Authors:** Alison Greig, Diana Dawes, Susan Murphy, Gillian Parker, Brenda Loveridge

**Affiliations:** 1 Department of Physical Therapy, Wesbrook Mall, The University of British Columbia, Vancouver, BC, Canada

**Keywords:** International health graduates, Educational program development, Program evaluation, Integration

## Abstract

**Background:**

The demand for health professionals continues to increase, partially due to the aging population and the high proportion of practitioners nearing retirement. The University of British Columbia (UBC) has developed a program to address this demand, by providing support for internationally trained Physiotherapists in their preparation for taking the National Physiotherapy competency examinations.

The aim was to create a program comprised of the educational tools and infrastructure to support internationally educated physiotherapists (IEPs) in their preparation for entry to practice in Canada and, to improve their pass rate on the national competency examination.

**Methods:**

The program was developed using a logic model and evaluated using program evaluation methodology. Program tools and resources included educational modules and curricular packages which were developed and refined based on feedback from clinical experts, IEPs and clinical physical therapy mentors. An examination bank was created and used to include test-enhanced education. Clinical mentors were recruited and trained to provide clinical and cultural support for participants.

**Results:**

The IEP program has recruited 124 IEPs, with 69 now integrated into the Canadian physiotherapy workforce, and more IEPs continuing to apply to the program. International graduates who participated in the program had an improved pass rate on the national Physiotherapy Competency Examination (PCE); participation in the program resulted in them having a 28% (95% CI, 2% to 59%) greater possibility of passing the written section than their counterparts who did not take the program. In 2010, 81% of all IEP candidates who completed the UBC program passed the written component, and 82% passed the clinical component.

**Conclusion:**

The program has proven to be successful and sustainable. This program model could be replicated to support the successful integration of other international health professionals into the workforce.

## Background

Health-care education around the world varies in educational standards, curriculum, and evaluation methods. The increasing demographic presence of ‘visible minorities’ and internationally-trained professionals, demands equal access to employment opportunities that support practice in occupational roles according to their qualifications and work experience. Support for this transition is essential to maximize skilled contribution to the development of a country [1]. Canada is known as a country with a broad immigration policy which is reflected in its ethnic diversity, with over 71,559 people transitioning from temporary to permanent Canadian resident status in 2010 [2]. The demand for health professionals continues to increase in Canada, partially due to the aging population and the high proportion of practitioners nearing retirement. Similar trends are predicted across all health professions [3-5], prompting the need for initiatives to increase the number of health professionals entering clinical practice. There is a need and desire to use readily available resources, the internationally-trained health professionals. To enter clinical practice, these health professionals need to demonstrate that they are safe and competent practitioners within the Canadian health care system. The knowledge and skills required to obtain a license to practice a profession vary by country and often by state or province, and most practitioners will require at least some orientation to the new health system and societal expectations of health care delivery. Others will require retraining in the basic sciences and clinical skills [6].

In order to obtain a license to practice Physiotherapy in Canada, in all provinces except Québec, Physiotherapists educated outside Canada must complete the two-part national Physiotherapy Competency Exam (PCE). In 2005 there was a notable discrepancy in pass rates between internationally trained Physiotherapists and Canadian graduates; less than 50% of internationally trained Physiotherapists exam attempts translated into passes, compared to greater than 92% of Canadian educated candidate attempts [7]. The challenges to internationally trained Physiotherapists success are thought to be lack of familiarity with the Canadian healthcare system, differences in Physiotherapy practice between countries, issues with language fluency, specialisation in specific areas of practice that provide a challenge on a generalist examination, and lack of familiarity with the Canadian National examination format.

There is some literature about internationally trained health professionals; this literature mainly reflects the process of examining the credentials, competency, demographic characteristics and distribution of international medical graduates (IMGs) [8-11]. There is some literature which considers IMG’s and nurse’s experiences while recertifying, or the training programs ability to aid integration into the recipient country’s medical community and the country’s needs [12-18]. The conclusions from this work are that medical training programs need: i) support for international graduates to facilitate program completion, including faculty and peer mentoring, psycho-social counselling, and educational and orientation activities; ii) help to master the contextual areas of practice in addition to ensuring academic and technical competence; iii) understanding from administrators and faculty of cross-national medical training and practices (in terms of similarities and differences) [18]. It is reasonable to suggest that the experiences and challenges experienced by IMGs and nurses as they recertify and integrate would be similar across other health professions.

To meet the demand for licensed Physiotherapists in Canada, and more specifically British Columbia (BC), and to provide support for internationally-trained physiotherapists who wish to continue to practice their skills and gain licensure, the “Internationally Educated Physiotherapists (IEP) Program Project” was established at the University of British Columbia.

## Methods

The aim of this project was to create the educational tools, curriculum, and infrastructure needed to sustain a program supporting internationally trained Physiotherapists applying for licensure in BC. The primary objective of the program was to increase the pass rate of internationally trained Physiotherapists taking the PCE. The UBC ethics board states that ethical approval is not needed for program evaluation.

### Program development

**Figure 1:**
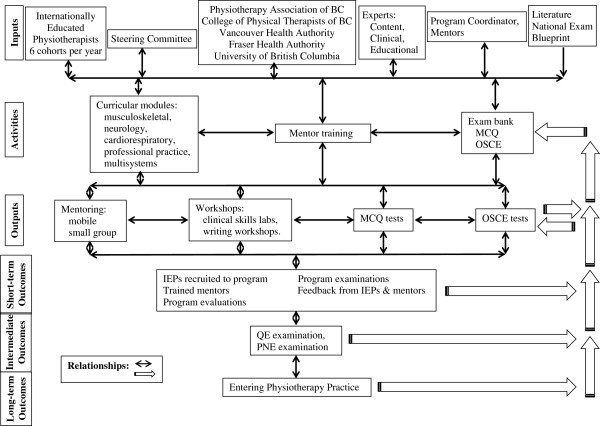
Logic model for Internationally Educated Physiotherapist (IEP) program.

The project team used a conceptual framework to map the program components, clarify the nature of program goals, and guide the program development [19]. To this end the program was developed using a logic model (Figure 1), which provides a schematic representation of the logical relationships involved in the transformation of resources into desired outcomes. The components of the logic model are: Inputs (the human, financial and material resources, and their organization); Activities (the services, products, and/or transactions that transform the inputs into outputs); Outputs (the transformation of resources into something that is delivered to clients); Outcomes (short-, intermediated, and long-term); Relationships (the relationships between the components) [20]. Finally, this project was evaluated using a “program evaluation” approach. Program evaluation has been defined as systematic investigation of the merit, worth, or significance of an object [21].

### Program implementation

The IEP project was developed in a partnership with key BC Physiotherapy stakeholders (university, professional organization, regulatory body, health authorities) which provided oversight, guidance, communication services, advertising and marketing support, identification and recruitment of mentors and content experts. A key role in the management of the program was the Program Coordinator, who was a trained and licensed Physiotherapist with over 40 years of practice experience. The Program Coordinator was responsible for all activities of the program, including recruiting, scheduling, data collection, communication and feedback sessions related to exam modules. All inputs and activities are shown in Figure 1. Initial estimates projected that the program could admit four IEPs per cohort and six cohorts per year, with some flexibility to include additional students if there was excess demand.

The program consists of two separate streams to assist IEPs in the preparation for the PCE: one in preparing for the written component (QE), and the other to prepare for the clinical component (PNE) of the national exam. IEPs had the option to participate in both streams of the program, or to receive support for the preparation of only one component of the PCE (the written (QE) component or the clinical (PNE) component). The QE consists of 200 multiple-choice questions (MCQs) and the PNE is an objective structured clinical examination (OSCE) to evaluate candidate’s interactions in controlled scenarios with standardised clients. The primary objective of the program is to assist IEPs with their preparation for taking these exams. To achieve this objective, modules including examination writing skills, content area review, and practice exam experiences (using tested-enhanced learning theory) were developed [22]. Many resources were available on-line; however, exams and workshops were initially delivered face-to-face.

Content experts developed initial versions of the curricular modules covering the four primary areas of Physiotherapy practice using the DACUM (Developing a Curriculum) model [23]. All modules were assessed by an external expert for accuracy, ease of use, and adherence to competencies as outlined by The Canadian Alliance of Physiotherapy Regulators 2008 National Examination Blueprint [24]. An updated version of the Alliance National Examination Blueprint was introduced in 2009 [25], that included a fifth area of practice (Multisystems); the IEP modules were revised to include this new area of practice. Two examination preparation modules were purchased from the national Physiotherapy Exam Skills Preparation Program and adapted to fit the IEP Program framework. Questions for the exam bank were developed by the content experts and reviewed by both a clinical expert and an educational expert to ensure the question format accurately reflected the questions found in the PCE.

Access to Physiotherapy mentors was initially identified as important to provide IEPs with the acquisition and reinforcement of practical skills in a Canadian context. Mentors were recruited from the clinical community based on area of clinical expertise, and experience teaching and supervising students. Three mentor-training workshops that focussed on facilitation techniques to support the learning of entry-level physiotherapy skills were offered to interested physiotherapists across the province. IEPs were matched with mentors who specialize in the primary areas of practice. The responsibilities of the mentors were to facilitate the development of the knowledge, clinical skills and clinical reasoning in each content area as expected in Physiotherapy practice in Canada. Mentors were also responsible for supporting the IEPs to achieve the competencies, as outlined in each module.

### Program evaluation

The program evolved since inception as a result of feedback from a variety of sources, including data collected from the IEPs, mentors and the IEP Advisory committee through focus groups and interviews. Feedback indicated that sample exam questions and practice exams were not perceived to accurately reflect the final PCE exam, and that there was a lack of detailed feedback after practice exams. Furthermore, IEPs suggested that modules were not specific enough and that IEPs were unfamiliar with terminology used within the Canadian Physiotherapy context. In response, study questions and a glossary of terms were added to each module, more complex vignette-based questions were developed, and based on the results of their exams IEPs are provided with guidance to further resources and study suggestions.

Several new initiatives resulted from the interviews with IEPs, including: clinical skills lab reviews; written tutorial workshops; mobile mentors; small group mentorship; delivery of written exam modules to remote communities; and transitioning the written program to an on-line platform. In addition, the written exam preparation was transitioned to a fully on-line program, so all aspects of the module was accessible to IEPs throughout BC, across Canada and even internationally.

### Program effectiveness

The first step of the Program Evaluation was the formation of a program logic model followed by analysis of the outcomes defined in the model. Data for IEP candidates, all international Physiotherapy graduates, and Canadian graduates is shown for the PCE across time (Table 1). The differences between National exam results for IEPs who had participated in the IEP Program compared with other internationally trained Physiotherapists who had not participated in the program were tested using Fisher’s exact test. The relative risk of passing the exams for IEPs and other internationally educated candidates was calculated, with 95% confidence interval using Wald’s formula. The term “risk” is used throughout the text as risk ratios were used rather than the term “chance” which implies other statistical tests.

**Table 1:** Canadian alliance of physiotherapy regulators examination results

	**2008**^**#**^	**2009**^**#**^	**2010**^**#**^	**2011**^**#**§^	Total*
**Written component (QE)**					
All candidates	738 (78)	755 (76)	766 (77)		2259 (77)
Canadian-educated	503 (93)	555 (94)	566 (94)		1624 (94)
Internationally-educated^+^	235 (58)	200 (49)	200 (51)		635 (53)
IEP program	1 (33)	7 (54)	21 (81)	13 (56)	43 (67)
**Clinical component (PNE)**					
All candidates	691 (85)	758 (85)	801 (88)		2250 (86)
Canadian-educated	506 (95)	538 (96)	618 (96)		1662 (96)
Internationally-educated^+^	185 (66)	220 (67)	183 (70)		588 (68)
IEP program	4 (100)	26 (100)	27 (82)	16 (80)	70 (92)

Data represent people who passed the exam (% pass).

^**#**^Year of taking exam.

*Since beginning 2008 to end Dec 2010.

^+^Includes IEP program candidates.

^§^Incomplete data (% is of known results), Alliance not published.

## Results

### Educational modular and curricular packages

Seven modules, based on the PCE blueprint and the core competencies of entry level physiotherapy in Canada, were developed and trialled.

### Exam bank

More than 500 MCQs and 22 OSCE questions were developed, reviewed and, stored in the exam bank with marking guides and patient model instructions where appropriate. This supported the administration of internal written tests and internal clinical tests.

### Mentorship

Initially 35 mentors were trained and paid for a maximum of 10 hours per IEP to provide one-on-one support and expertise during the exam preparation. The number of mentors trained was sufficient for the number of IEPs residing in the Lower Mainland of British Columbia, however, there were challenges pairing mentors with IEPs who resided out of the Lower Mainland, particularly those IEPs in rural areas. To address this, mobile and small group mentorship models were developed, which involved mentors from the Lower Mainland travelling to meet with IEPs in remote areas. This model proved to be cost effective and time efficient. In addition, higher IEP to mentor ratios (small group learning), maximised the mentor time and provided a more “dynamic” session for the IEPs. Feedback from IEPs and mentors, indicated that few IEPs utilized the allocated time with mentors, and that IEPs found the other components of the program (e.g. practice exams, workshops, clinical lab days etc.) of more benefit. The mentorship model therefore moved to self-regulated access; that is, IEPs were given contact details for mentors and were encouraged to make independent arrangements to meet with mentors according to need.

### IEP candidates

**Figure 2:**
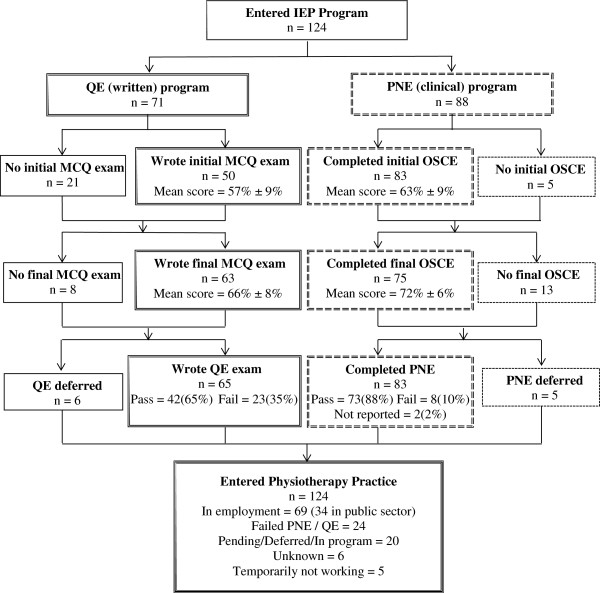
Flow diagram of candidates through Internationally Educated Physiotherapist (IEP) Program (July 2008 to January 2012).

The first cohort of nine IEPs was admitted into the program in July 2008. Figure 2 shows the flow of IEPs through the program. As of 1st January 2012, 124 IEPs have participated in the program. Data on IEP characteristics, Table 2, demonstrate that IEPs come from a variety of countries, with the majority coming from the UK (31%) and India (21%). Most IEPs have been employed as a Physiotherapist for less than five years (62%), with 14% never having practiced as a Physiotherapist prior to entering the program. In the first three years, more than 65% of the IEPs had English as their first language but this dropped to 50% in 2011.

**Table 2:** Characteristics of international educated physiotherapists accepted into program

	2008*	2009*	2010*	2011*	**Total**
**Number admitted**	13	31	38	42	124
**Sex nMale (%Male)**	10 (77)	15 (48)	18 (47)	16 (38)	59 (48)
**Years of practice as physiotherapist**					
Never practiced	1 (8)	11 (35)	6 (16)	0	18 (14)
<5	8 (61)	16 (52)	21 (55)	15 (36)	60 (48)
5 to 10	3 (23)	4 (13)	7 (18)	22 (52)	36 (29)
>10	1 (8)	0	1 (3)	5 (12)	7 (6)
Not recorded	0	0	3 (8)	0	3 (2)
**Country of physiotherapy education**					
Australia	1 (8)	6 (19)	7 (18)	1 (2)	15 (12)
Austria	0	0	1 (3)	0	1 (1)
Belgium	0	0	0	1(2)	1(1)
Brazil	2 (15)	0	1 (3)	3 (7)	6 (5)
Egypt	0	0	0	1 (2)	1 (1)
Finland	1 (8)	0	0	0	1(1)
India	0	3 (10)	6 (16)	17 (40)	26 (21)
Iran	0	1 (3)	2 (5)	2 (5)	5 (4)
Israel	0	0	1 (3)	3 (7)	4 (3)
Netherlands	1 (8)	1 (3)	2 (5)	0	4 (3)
New Zealand	1 (8)	1 (3)	1 (3)	0	3 (2)
Pakistan	0	0	1 (3)	0	1 (1)
Philippines	0	0	2 (5)	7 (17)	9 (7)
South Africa	1 (8)	0	0	1 (3)	2 (2)
United Kingdom	6 (46)	18 (58)	9 (24)	5 (12)	38 (31)
United States	0	1 (3)	4 (10)	1 (2)	6 (5)
Unknown	0	0	1 (3)	0	1 (1)
**English as 1st language**	10 (77)	28 (90)	25 (66)	21 (50)	84 (68)

All data are n (%). Percentages do not always add up to 100% due to rounding.

*Year of entering program.

### Written and clinical exams

Table 1 shows the PCE exam results for IEP program participants relative to all PCE candidates, Canadian-educated candidates, and all Internationally-educated candidates examination results. In the first year, 2008, there were very few graduates from the IEP program due to timing of the exams, three taking the written (QE) component and four the clinical (PNE) component, with 33% and 100% passing respectively. In 2010, 26 IEPs took the QE exam with 81% passing and, 33 took the PNE with 82% passing. The pass rate for IEPs taking the QE between 2008 and 2012 is 0.67, and for those taking the PNE is 0.92. These rates are significantly higher (p < 0.05 and p < 0.001, respectively using Fisher’s exact test) than the pass rate for all the internationally-educated physiotherapists who took the QE and PNE where the pass rate is 0.53 and 0.68 respectively. Taking the IEP program conveys a 28% increase in risk of passing the written exam, with a risk ratio (RR) in comparison to other international graduates taking the QE of 1.28 (95% CI, 1.02 to 1.59). The results from the PNE are even more positive with RR = 1.39 (95%CI, 1.27 to 1.52); IEP candidates have a 39% increase in risk of passing the clinical exam when compared to international candidates who do not take the IEP program.

Of the 65 IEPs taking the QE exam 65% had English as their first language, of these 63% passed the exam. Of the 35% who did not have English as their first language, 40% passed the QE. IEP candidates who have English as their first language have a non-statistically significant 58% increased risk of passing the QE, RR = 1.58 (95% CI 0.93 to 2.67, p = 0.09). Of the 83 IEPs taking the PNE exam, 74% had English as their first language, of these 93% passed the exam. Of the 26% who did not have English as their first language, 67% passed the PNE. For the PNE, IEP candidates who have English as their first language have a statistically significant, 40% increased risk of passing with an RR = 1.4(95% CI, 1.03 to 1.9, p = 0.03).

Of the 124 people who entered the IEP program 69 are known to have entered the Canadian physiotherapy workforce, with 34 of these known to be working in the public sector, five people are temporarily not in the Canadian workforce, due to child care or visa problems, and 20 are awaiting immigration approval for entry to Canada.

## Discussion

The aim of the IEP Program was to create the educational tools and infrastructure needed to sustain a program supporting internationally trained Physiotherapists applying for licensure in BC. Consideration was given to developing a program that included the necessary curriculum, resources, mentorship and practice opportunities to assist IEPs in their preparation for the exams and entering the Canadian workforce. As many resources as possible were made available on-line to serve the needs of IEPs from clinical practice sites distant from Vancouver. Evaluation was built into the program to inform continuing program improvements and the development of additional program initiatives. This has resulted in the Program attracting greater numbers of IEPs than projected, and providing the flexibility to support and accommodate these greater numbers. Projections identified the capacity to support and accommodate 8 IEPs per year in the practical and 16 in the written, and over the first 4 years of the program (2008–2011), 124 IEPs have participated. The program now recovers its expenses through charging the IEPs, and although participation has declined relative to the funded program, enrolment has been sufficient to cover costs. Using a framework to build the modules, gaining regular feedback and, making the necessary changes to develop an internationally educated health professional program has proven highly successful. Only 24 health professionals (19%) who entered the program were unable to succeed in national examinations and join the Canadian physiotherapy workforce. However, these professionals may retake the examination and enter the workforce in the future. The long-term data regarding entry of IEPs into practice indicates that at least 69 IEPs, who completed the IEP Program, entered the Canadian Physiotherapy workforce, with 34 of these working in the public sector.

The IEPs who participated appear to be representative of the population of Canadian immigrants; in the Canadian general populace the ratio of European to Asian immigrants is changing over time. In our population 58% of students came from the UK in 2009 and 12% in 2011, versus 10% of students from India in 2009 and 40% in 2011. The top four countries for physiotherapist immigrants practicing in Canada to have received their basic physiotherapy education, from 2007 to 2009, were UK (21%), India (15%), USA (10%) and Australia (8%) [26]. The IEP program had fewer applicants from the U.S.A. and more from Australia; this could be explained by British Columbia having a lower U.S.A. and higher immigration rate from Asia and the Pacific than other provinces (Canadian average = 9% from USA, 25% from Asia and the Pacific; B.C. 6% and 59% respectively). Training, examination methods, healthcare context, and physiotherapist roles in the UK and Australia align more closely with Canadian standards than those from other countries (i.e. India and the Philippines); it is possible that Physiotherapists from countries with different academic procedures seek more opportunities to receive additional support for the exam preparation.

The primary objective of this program was to increase the pass rate of internationally trained Physiotherapists taking the PCE. The success of the program in increasing the pass rate of IEPs taking the PCE has been achieved with international graduates who took the program having a 28% greater possibility of passing the written section than their counterparts who did not take the program. The results for the clinical exam are even more impressive with the IEP program students having a 39% increase in risk of passing. A greater percentage of Canadian-educated candidates for the PCE continue to pass both elements of the PCE but the gap is closing, particularly in the clinical component. The program was developed partially due to the observation that in 2005 less than 50% of PCE attempts translated into passes in IEPs [7]; the students who participate in the program now far surpass this with more than 65% gaining a pass. Trends in IEP successes on the QE also indicate higher pass rates over time, suggesting a positive impact of the new initiatives including the written workshops, additional resources (expanded exam bank, additional written exam practice, glossaries and self-study questions), mobile mentors, small group mentorship, and transitioning the written program to an on-line platform to promote access throughout BC, across Canada and internationally.

It may be expected that those who do not have English as their first language would have more difficulty with participating in the course and passing the exams, in particular, the written element. This appears to be false for the QE, the relative risk is 1.58 but the confidence interval is very wide, crossing 1 (equality) and is not significant. However, in the slightly larger number of people taking the PNE (n = 81), the relative risk demonstrates that those with English as their first language have a significant, 40% greater risk of passing the clinical element. The IEP success rate on the PNE indicates a slight decrease over time, when more candidates who did not have English as their first language participated.

Although the IEP program targets internationally trained Physiotherapists, this model could be transferred to other health professions given the identified need to support internationally trained health professionals transitioning into the Canadian workforce [14], and the similarity in processes related to the passing competency examinations prior to registration in these professions [27]. Using program evaluation methodology, including the logic model, proved a successful approach for the development of a program that aimed to integrate internationally educated health professionals into clinical practice.

### Limitations

As this is a relatively new program, there are not sufficient numbers of IEPs who have participated to enable sub-analyses of the data and examination of particular elements of the program. Collecting more qualitative data will allow for more in-depth analysis of the IEPs integration into Canadian practice and the impacts on acculturation and socialization.

## Conclusions

Potentially this program model could be applied to support integration of international health graduates from any background into the workforce. While this program used as a primary outcome measure improved performance on national competency examinations, many program elements also support international practitioner’s integration into the Canadian health care system and practice. Further examination of the role specific program elements may have on improving internationally educated health professional’s understanding of the cultural context of Canadian practice is needed.

## Competing interests

GP received reimbursement for her work coordinating the IEP program.

## Authors’ contributions

AG conceived of the study and participated in its design and coordination and helped to draft the manuscript. DD carried out the analyses and drafted the manuscript. SM participated in study design and coordination. GP participated study coordination. BL oversaw study concept, design and coordination. All authors read and approved the final manuscript.

## Pre-publication history

The pre-publication history for this paper can be accessed here:

http://www.biomedcentral.com/1472-6920/13/140/prepub
